# Investigation of hemispheric asymmetry in Alzheimer’s disease patients during resting state revealed by fNIRS

**DOI:** 10.1038/s41598-024-62281-y

**Published:** 2024-06-11

**Authors:** Hazel Gül Mızrak, Merve Dikmen, Lütfü Hanoğlu, Bayram Ufuk Şakul

**Affiliations:** 1 Department of Anatomy, School of Medicine, Istanbul Medipol University, Istanbul, Turkey; 2 Regenerative and Restorative Medicine Research Center (REMER), Research Institute for Health Sciences and Technologies (SABITA), Istanbul Medipol University, Istanbul, Turkey; 3 Program of Electroneurophysiology, Vocational School of Health Services, Istanbul Medipol University, Istanbul, Turkey; 4 Department of Neurology, Istanbul Medipol University Training and Research Hospital, Istanbul, Turkey

**Keywords:** Nervous system, Brain, Neuroscience, Cognitive ageing, Cognitive neuroscience, Neurology, Neurological disorders, Dementia, Alzheimer's disease, Imaging and sensing

## Abstract

Alzheimer's disease (AD) is characterized by the gradual deterioration of brain structures and changes in hemispheric asymmetry. Meanwhile, healthy aging is associated with a decrease in functional hemispheric asymmetry. In this study, functional connectivity analysis was used to compare the functional hemispheric asymmetry in eyes-open resting-state fNIRS data of 16 healthy elderly controls (mean age: 60.4 years, MMSE (Mini-Mental State Examination): 27.3 ± 2.52) and 14 Alzheimer's patients (mean age: 73.8 years, MMSE: 22 ± 4.32). Increased interhemispheric functional connectivity was found in the premotor cortex, supplementary motor cortex, primary motor cortex, inferior parietal cortex, primary somatosensory cortex, and supramarginal gyrus in the control group compared to the AD group. The study revealed that the control group had stronger interhemispheric connectivity, leading to a more significant decrease in hemispheric asymmetry than the AD group. The results show that there is a difference in interhemispheric functional connections at rest between the Alzheimer's group and the control group, suggesting that functional hemispheric asymmetry continues in Alzheimer's patients.

## Introduction

The human brain is divided into two hemispheres: the left and the right hemisphere. In adults, these hemispheres are not mirror images and exhibit numerous anatomical and morphological asymmetries^1^. The left and right hemispheres have distinct functional differences, with the left hemisphere being dominant in language processing, as first reported^2^. The molecular and genetic basis of the brain hemispheres' anatomical and functional asymmetry is not fully understood, despite its well-known existence^3^.

Alzheimer's disease (AD) is a neurodegenerative disorder characterized by the destruction of neurons, resulting in dementia with a gradual loss of memory, judgment, and ability to function. This disorder typically affects individuals over the age of 65, although less common forms of the disease can also occur in early adulthood^4,5^. The development of Alzheimer's is believed to be contributed to by several brain changes, including the deposition of beta-amyloid protein (also known as beta-amyloid plaques) outside neurons and the accumulation of an abnormal form of tau protein (also known as tau tangles) inside neurons^6^. Especially, Alzheimer's disease is characterized by the deposition of beta-amyloid (Ab) plaques in specific regions of the brain^7^. In AD, beta-amyloid and tau proteins accumulate in the brain, leading to inflammation, neuronal atrophy, and cell death^8^. Brain atrophy in AD often occurs asymmetrically and is associated with the loss of normal brain asymmetries during development and aging^5^. Previous studies have shown that cortical thinning, amyloid-beta plaques, and disruption of neurite connectivity are more pronounced in the left hemisphere of Alzheimer's patients^9^. A recent study found that cortical thinning thins asymmetrically throughout adult life, with this thinning being more accelerated in AD compared to healthy aging^10^. In a separate study, researchers discovered rightward asymmetry in the white matter networks of individuals with AD. They proposed that this rightward asymmetry was due to impairments in the left hemisphere^11^. However, the literature still preserves the mystery of functional hemispheric asymmetry in AD.

Research has shown that prefrontal cortex (PFC) activity during cognitive tasks is less lateralized in older adults compared to younger adults. This phenomenon is commonly referred to as Hemispheric Asymmetry Reduction in Older Adults (HAROLD) and has been explained through a model of the same name^12^. The HAROLD model considers the common findings of multiple studies^13–17^. Cabeza et al. conducted a study investigating the effects of aging on brain activity during word encoding and recall. The study found that prefrontal cortex (PFC) activity in young adults is left-lateralized during encoding and right-lateralized during recall. In contrast, older adults exhibit less PFC activity during encoding and bilateral PFC activity during recall^17^. Cabeza R. interpreted studies that obtained bilateral activation in older adults as a decrease in hemispheric asymmetry and gathered them under a common model, defining them as the HAROLD model. According to the HAROLD model, there have been differing opinions on whether age-related decreases in lateralization serve a purpose or are merely a consequence of aging's impact on the brain. In 1997, Cabeza et al.^18^ published a study suggesting that reductions in asymmetry may play a compensatory role in the aging brain, according to the Compensation View^12^.

According to Dolcos et al.'s hypothesis on right hemisphere aging, age-related cognitive declines affect functions attributed to the right hemisphere more than those associated with the left hemisphere. This asymmetry is related to the type of information processed by each hemisphere. The left hemisphere is primarily involved in processing verbal information, while the right hemisphere is primarily involved in processing pictorial and spatial information. When considering these findings collectively, it is suggested that the right hemisphere is more impacted by the reduction in hemispheric asymmetry and lateralization that occurs with age^3^.

Functional Near-Infrared Spectroscopy (fNIRS) is a non-invasive optical imaging method^19^. A study analyzing hemispheric asymmetry using NIRS found that NIRS-based connectivity was more sensitive and provided more comprehensive information compared to traditional activation. This could help us better understand the functional organization of the brain^20^. The literature states that the fNIRS method enables the measurement of functional connectivity in tasks such as finger movement^21^, Stroop task^20^, and during the resting state^22^. Furthermore, the literature reports that functional connectivity can be measured in pain sensation processes using a sensory stimulus^23^. Additionally, some studies have shown that this imaging method can detect spontaneous hemodynamic fluctuations^24,25^. It is reported in the literature that dementia, including mild cognitive impairment (MCI) and AD, is characterized by changes in cerebral blood flow. Most studies have reported changes in resting cerebral oxygenation levels, fluctuations, or connectivity in patients with Alzheimer's and Mild cognitive impairment^26^. A recent study has shown that fNIRS is a suitable technology for detecting AD and monitoring its progression^27^.

Recent advances in brain functional network analysis have revealed evidence that the brain's functional architecture is relatively stable at rest as well as during various cognitive tasks^28^. Resting-state functional connectivity measures temporal correlations in spontaneous fluctuations between spatially distributed brain regions and is increasingly used for fNIRS^29^. Furthermore, Medvedev's published study revealed hemispheric asymmetry of functional connectivity in the resting state^28^.

The aim of our study is to compare the decrease in age-related hemispheric asymmetry in people diagnosed with AD and healthy elderly individuals according to the hemodynamic responses of the brain and to provide a new perspective on both early diagnosis and treatment methods of AD in the clinic by revealing their differences.

## Materials and methods

### Participants

16 healthy subjects (mean age: 60.4 years, MMSE (Mini-Mental State Examination): 27.3 ± 2.52) and 14 patients diagnosed with AD (mean age: 73.8 years, MMSE: 22 ± 4.32) were included in the study. Patients with AD were recruited from the Department of Neurology at Bagcılar Medipol Mega University Hospital and were diagnosed by an experienced neurologist based on the National Institute of Neurological and Communicative Diseases and Stroke/Alzheimer's Disease and Related Disorders Association (NINCDS-ADRDA) diagnostic criteria^30^. Inclusion criteria for controls and AD patients in this study included: (1) age 60–80, (2) completed primary school. General exclusion criteria were alcohol/substance use, sinistrality (all participants were right-handed according to the Edinburgh Handedness Questionnaire), and current and chronic untreated neurological or psychiatric illness.

The study was conducted in accordance with the principles of the Declaration of Helsinki. Participants voluntarily provided written informed consent without any form of compensation offered for their involvement, as explicitly outlined in the informed consent documentation. The study was approved by the Istanbul Medipol University Ethics Committee (No: 10840098-604.01.01-E.25335).

### Data acquision

Functional Near-Infrared Spectroscopy (fNIRS) is a non-invasive optical imaging method that records the brain's release of oxygenated (O2Hb) and deoxygenated (HHb) hemoglobin associated with neuronal activity via the Blood Oxygen Level Dependent (BOLD) effect. This scalp-based technique is objective and provides precise measurements of brain activity. This technique is portable, relatively inexpensive, lightweight, and resistant to motion artifacts of a mechanical nature, like electroencephalography (EEG). Therefore, it is suitable for ecological measurements during the implementation of clinical tests^31^.

**Figure 1:**
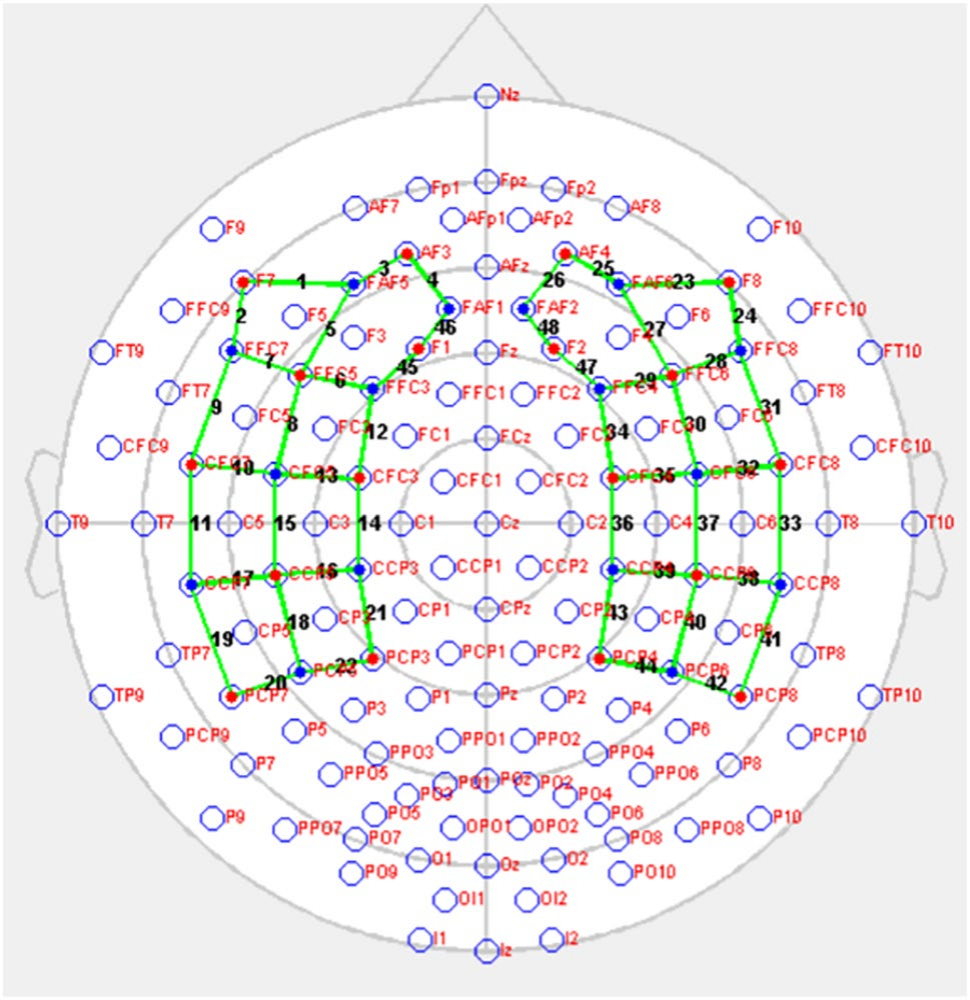
The fNIRS Data Acquisition Head Surface Distribution (Red: fNIRS Source Optodes, Blue: fNIRS Detector Optodes, Green: fNIRS channels). The homologous channel pairs between the two hemispheres are as follows: ch 1–ch23, ch 2–ch 24, ch 3–ch 25, ch 4–ch 26, ch 5–ch 27, ch 6–ch2 9, ch 7–ch 28, ch 8–ch 30, ch 9–ch 31, ch 10–ch 32, ch 11–ch 33, ch 12–ch 34, ch 13–ch 35, ch 14–ch 36, ch 15–ch 37, ch 16–ch 39, ch 17–ch 38, ch 18–ch 40, ch 19–ch 41, ch 20–ch 42, ch 21–ch 43, ch 22–ch 44, ch 45–ch 47, ch 46–ch 48. (ch is used as channel abbreviation).

The changes in the oxyhemoglobin (HbO), deoxy-hemoglobin (HbR) and total-hemoglobin (tHb) concentrations were recorded using fNIRS system (NIRx Medical Technologies LLC, Berlin, Germany) from 34 optodes with 18 wavelengths (760 and 850 nm) laser sources and 16 detectors for each hemisphere. 48 optical channels (24 for each hemisphere) were acquired (Fig. 1). The channels were located in the temporoparietal region and inferior frontal regions, where significant results have been reported in the literature^32^. Subjects were instructed to sit in a chair, to focus their gaze on the crosshair in the center of the computer monitor to minimize eye movements and not to think about anything during a resting period of 4 min. Signals were pass band filtered between 0.04 and 0.1 Hz. NIRStar software (NIRStar, 15.2, 2018, https://nirx.net/software) was used to obtain fNIRS data and to check the signal quality before recording. Subjects who could not obtain data in sufficient time and quality were excluded from the study. Thus, fNIRS data from 14 subjects in the AD group and 16 subjects in the control group were included in the study.

### Data analyses

Analysis of the fNIRS data was performed using the software package NIRS-KIT (NIRS-KIT V3 Beta, 2023, https://www.nitrc.org/projects/nirskit/) implemented in MATLAB (MathWorks, Inc., Natick, MA, USA) developed by Hou et al.^29^.

**Figure 2:**
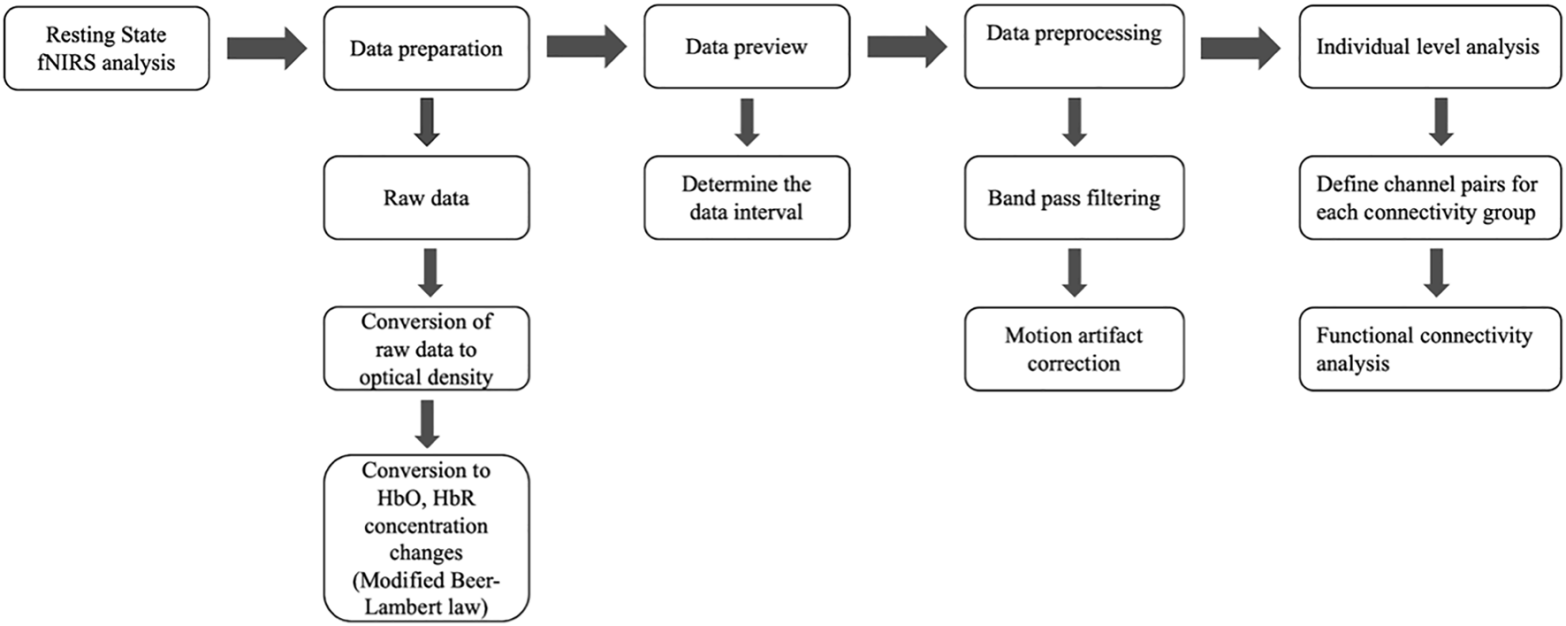
Diagram of the data processing pipeline of resting-state fNIRS data.

Data obtained from fNIRS devices are converted into HbO and HbR concentrations via the Modified Beer-Lambert law in the NIRS-KIT supported format. 4 min (240 s) data obtained from subjects were processed into NIRS-KIT. The raw data we collected included HbO2, Hb, and HbT. In our study, we focused only on changes in HbO concentration. Raw fNIRS time series data were converted to optical density (OD) using the modified Beer-Lambert law. The TDDR (Temporal derivative distribution repair) method was used for motion correction. The TDDR (Temporal Derivative Distribution Repair) method was employed for motion correction. This method utilizes an adaptive motion artifact reduction algorithm, iteratively allocating weights to the temporal derivatives of the ΔHbO signal and reconstructing the signal through the integration of these weighted derivatives^33^. Bandpass filtered within IIR (Infinite Impulse Response) range of 0.04–0.1 Hz. This bandpass filtering was used to remove noise and physiological factors such as respiration and heart oscillation^22,34^. After the preprocessing, the functional connectivity analysis was performed. Resting-state functional connectivity measures temporal correlations in spontaneous fluctuations between spatially distributed brain regions^29^ (Fig. 2).

The Pearson's correlation coefficient between the time series of all channels was calculated to obtain a 24 × 24 connection matrix for each subject. Previous studies have shown that the biological explanation for the negative correlation is ambiguous, thus we only focused on the positive correlation^35,36^. Fisher r-to-z method was used to convert the correlation coefficients to *z* values to improve normality.

### Statistical analysis

All statistical analyses were performed using SPSS statistics software (IBM SPSS Statistics, Sofware version: 20.0, 2021, https://www.ibm.com/support/pages/downloading-ibm-spss-statistics-20). The Shapiro–Wilk normality test was performed for demographic differences in age, education, sex and Mini-Mental State Examination (MMSE) scores. Independent t test was used to normally-distributed parameters and The Mann–Whitney U-test was used to nonnormally-distributes parameters.

Independent sample t-test (Welch’s t test) was performed to determine the effect of interhemispheric coherence on groups for each channel pair that located in parallel in the two hemispheres and paired sample t-test was performed to compare the intrahemispheric adjacent channels with those in the opposite hemisphere for each group separately. Functional connectivity analyses were calculated using Fisher's z-score. Significance levels were determined as p < 0.05. Linear regression analysis was performed to assess the effect of age and sex on functional connectivity.

## Results

### Demographic and clinical results

Functional connectivity analysis was performed on the resting-state fNIRS datas of 14 subjects in the Alzheimer's group and 16 subjects in the control group, and the statistical results were reported. According to the Shapiro–Wilk normality test performed to demographic differences, MMSE and sex were not normally distributed (p > 0.05). Table 1 shows the demographic and clinical characteristics of the two groups.

**Table 1:** Demography and MMSE score of control subjects and AD patients (p values indicate normal distribution test values).

	Age	Sex (Female)	MMSE	Education
AD group (n:14)	73.8 ± 7.12	3 (21.4)	22(21–25)	14.2 ± 5.84
Control group (n:16)	60.4 ± 6.32	6 (37.5)	28(26–29)	10.8 ± 5.81
p values	< 0.001	0.039	< 0.001	0.136

Normally distributed variables were presented as mean ± SD, non-normally distributed continuous variables were presented as median (Q1-Q3), categorical variables were presented as counts (percentages).

### Comparison of functional connectivity between the two hemispheres across groups

#### HbO concentrations

**Figure 3:**
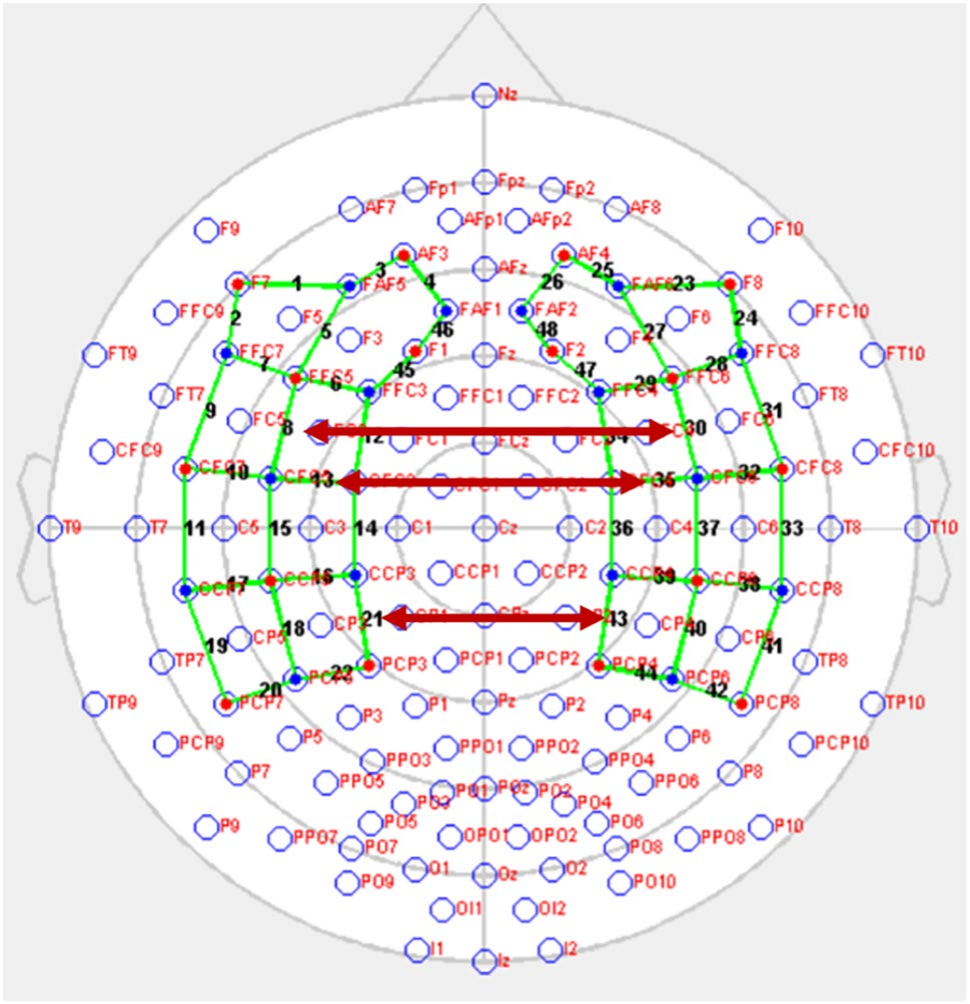
Homologous channels with significant differences detected in the comparison of functional connectivity between the two hemispheres across groups (HbO).

The functional connectivity analysis revealed significant differences between the two groups in several brain regions. Specifically, homologous channels in the premotor and supplementary motor cortex (BA6) (ch 8–ch30) [t(25,53) = − 2.335, p = 0.028, *uncorrected*], primary motor cortex (BA4 and 3, 1,2) (ch 13–ch 35) [t(28) = − 2, 064, p = 0.048, *uncorrected*], and inferior parietal cortex and primary somatosensory cortex (BA39-40) (ch 21–ch 43) [t(26,25) = − 2.298, p = 0.030, *uncorrected*] (Fig. 3). We employed a linear regression analysis to assess the relationship between age and sex and the interhemispheric connectivity. Significant positive relationship between age, sex and ch 13–ch 35 (*r* = 0.240, *p* = 0.025, *uncorrected*) and ch 21–ch 43 (*r* = 0.207, *p* = 0.044, *uncorrected*). No other comparisons were significant. This indicates that 20% of the variance in interhemispheric connectivity is explained by differences in age and sex.

**Figure 4:**
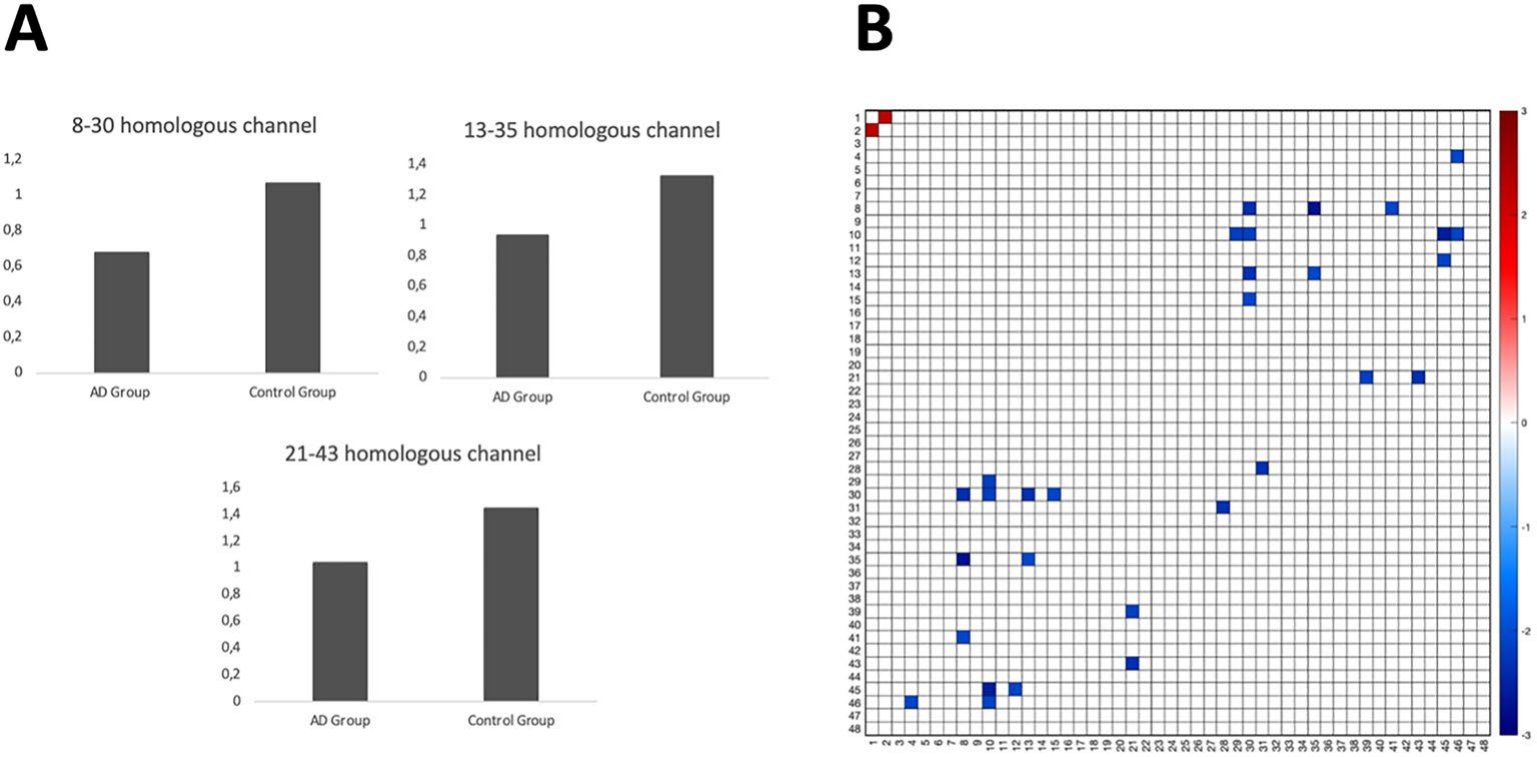
(**A**) Result of the statistical analysis using independent t test. Bars show the interhemispheric correlation data normalized to the z score by using Fisher’s z transformation (HbO). (**B**) The resting-state functional connectivity in oxyhemoglobin. Channel connections with significant inter-group differences (p < 0.05, *uncorrected*).

The anatomical localizations of the channels with significant differences were determined using the AAL2 brain atlas in the 'fNIRS optodes' location decider (fOLD)' toolbox (Supplementary Table S1)^37^. This significant difference between homologous channels indicated that the functional connectivity (in other words, communication) of these brain regions was weaker in AD group than control group (AD group showed weaker interhemispheric functional connectivity than control groups) (Fig. 4A).

Matrix image of comparison of the functional connectivity of fNIRS channels in the two hemispheres between the two groups (HbO) was made through the NIRS-KIT^29^ analysis program. This image matrix focuses on the regions representing channels numbered ch. 8–30, ch. 13–35, and ch. 21–43, where a significant difference was observed (Fig. 4B).

### HbR concentrations

**Figure 5:**
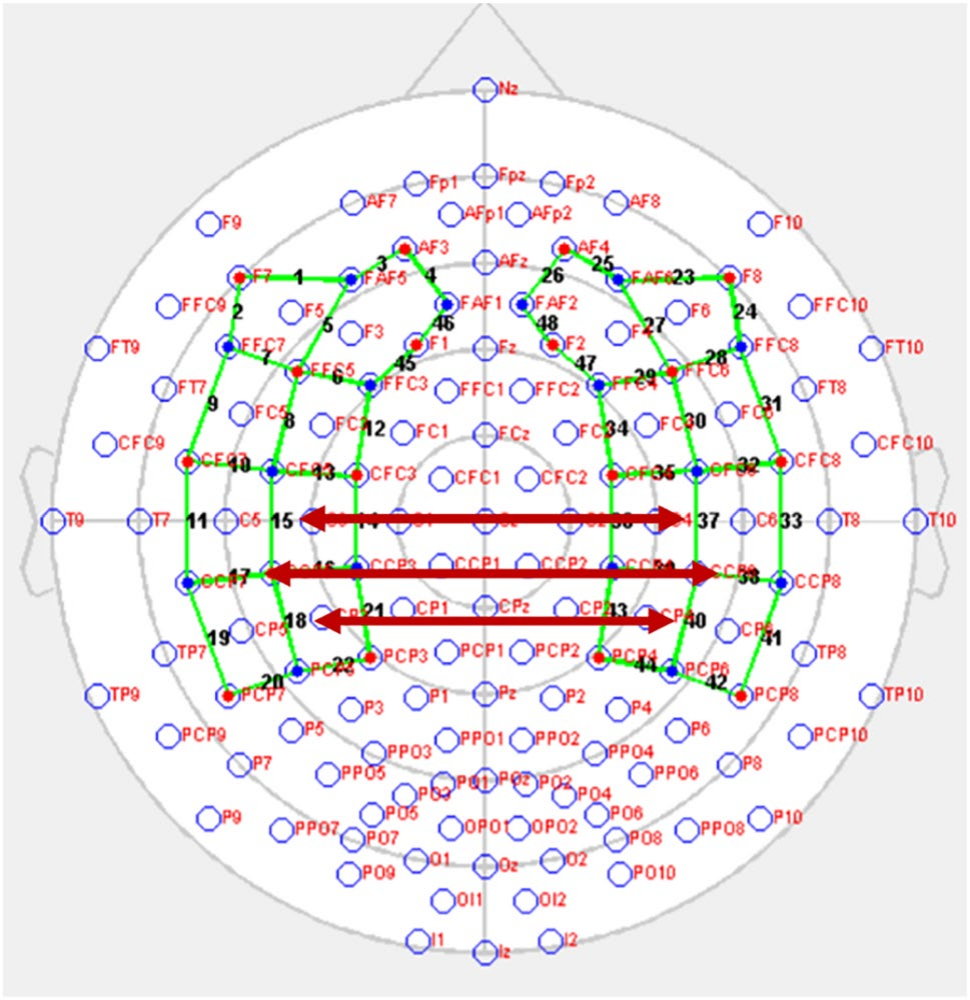
Homologous channels with significant differences detected in the comparison of functional connectivity between the two hemispheres across groups (HbR concentration).

The functional connectivity analysis revealed significant differences between the two groups in several brain regions. Specifically, homologous channels in the primary motor cortex and the primary somatosensory cortex (BA4 and 3,1,2) (ch 15–ch 37) [t(27,815) = − 2.552, p = 0.017, *uncorrected*], inferior parietal cortex (BA39-40) (ch 18–ch 40) [t(27,260) = − 2.104, p = 0.045, *uncorrected*], and Supramarginal gyrus (ch 17–ch 38) [t(23,371) = − 2.641, p = 0.015, *uncorrected*] (Fig. 5). We employed a linear regression analysis to assess the relationship between age and sex and the interhemispheric connectivity. No comparisons were significant (p > 0.05, *uncorrected*).

**Figure 6:**
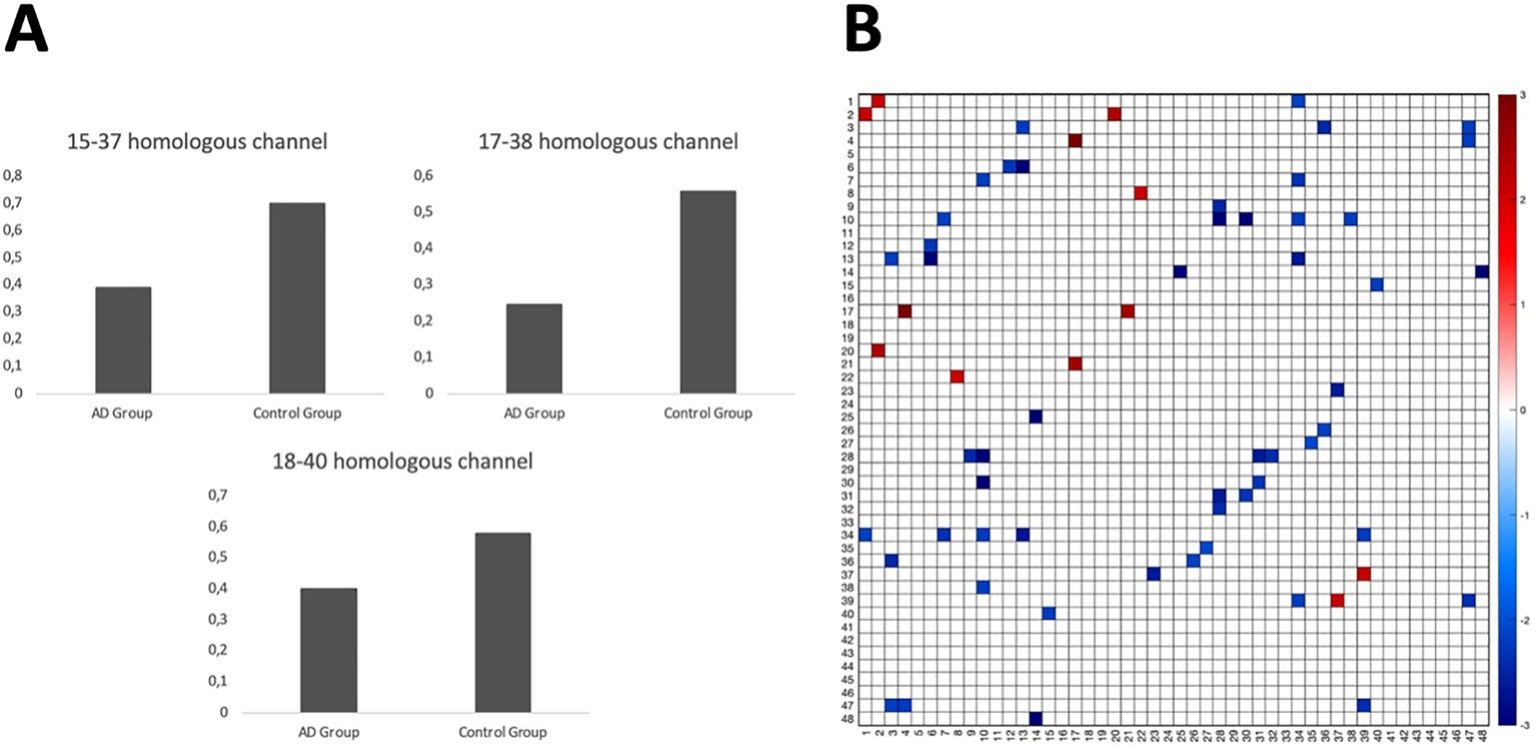
(**A**) Result of the statistical analysis using independent t test. Bars show the interhemispheric correlation data normalized to the z score by using Fisher’s z transformation (HbR). (**B**) The resting-state functional connectivity in deoxyhemoglobin. Channel connections with significant inter-group differences (p < 0.05, *uncorrected*).

The anatomical localizations of the channels with significant differences were determined using the AAL2 brain atlas in the 'fNIRS optodes' location decider (fOLD)' toolbox^37^. This significant difference between homologous channels indicated that the functional connectivity (in other words, communication) of these brain regions was weaker in AD group than control group (AD group showed weaker interhemispheric functional connectivity than control groups) (Fig. 6A).

Matrix image of comparison of the functional connectivity of fNIRS channels in the two hemispheres between the two groups (HbR) was made through the NIRS-KIT^29^ analysis program. This image matrix focuses on the regions representing channels numbered ch. 15–37, ch. 17–38, and ch. 18–40, where a significant difference was observed (Fig. 6B).

### Comparison of intrahemispheric functional connectivity within group

#### HbO concentrations

Intrahemispheric adjacent channels of the channels showed significant effect across groups were determined. The new channel (ch.) pairs were identified as interhemispheric adjacent channel pairs, separately in the right and left hemispheres. Channels 8, 13, and 21 were located in the left hemisphere, while channels 30, 35, and 43 were located in the right hemisphere. Intrahemispheric adjacent channels of channel 8 are ch. 6, ch.7, ch.9, ch.10, ch. 12, ch. 13. Intrahemispheric adjacent channels of channel 30 are ch. 28, ch. 29, ch. 31, ch. 32, ch. 34, ch. 35. Intrahemispheric adjacent channels of channel 13 are ch. 6, ch. 8, ch. 12, ch. 14, ch. 15, ch. 16. Intrahemispheric adjacent channels of channel 35 are ch. 29, ch. 30, ch. 34, ch. 36, ch. 37, ch. 39. Intrahemispheric adjacent channels of channel 21 are ch. 16, ch. 18, ch. 22. Intrahemispheric adjacent channels of channel 43 are ch. 39, ch. 40, ch. 44. The new channel pairs analyzed were as follows: 13–12 with 35–34 (1. Adjacent ch. pairs), 13–6 with 35–29 (2. Adjacent ch. pairs), 13–8 with 35–30 (3. Adjacent ch. pairs), 13–14 with 35–36 (4. Adjacent ch. pairs), 13–16 with 35–39 (5. Adjacent ch. pairs), 13–15 with 35–37 (6. Adjacent ch. pairs), 21–16 with 43–39 (7. Adjacent ch. pairs), 21–18 with 43–40 (8. Adjacent ch. pairs), 21–22 with 43–44 (9. Adjacent ch. pairs), 8–7 with 30–28 (10. Adjacent ch. pairs), 8–9 with 30–31 (11. Adjacent ch. pairs), 8–10 with 30–32 (12. Adjacent ch. pairs), 8–13 with 30–35 (13. Adjacent ch. pairs), 8–12 with 30–34 (14. Adjacent ch. pairs), 8–6 with 30–29 (15. Adjacent ch. pairs).

**Figure 7:**
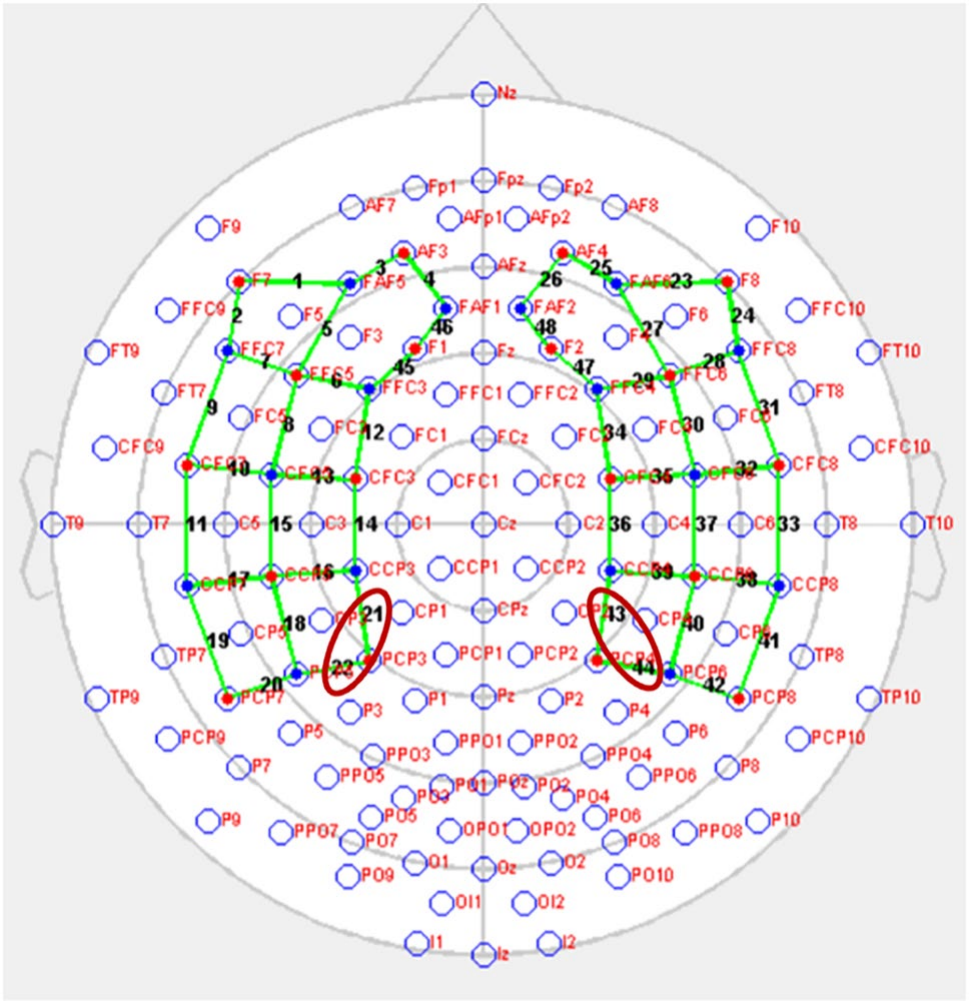
Adjacent channels with significant differences in comparison of interhemispheric functional connectivity within the group (HbO concentration).

To analyze the functional connectivity of adjacent channel pairs, we performed a paired sample t-test separately for the AD and control groups. The statistical analysis revealed a significant difference in the functional connectivity analysis outputs of intrahemispheric adjacent channel pairs numbered 21–22 and 43–44 in the control group [t(15) = 2.380, p = 0.031, *uncorrected*] (Fig. 7). This significant difference indicated that the functional connectivity (in other words, communication) between these regions was different between the two hemispheres in control group. The left hemisphere channels^21,22^ showed stronger correlation values for functional connectivity than the right hemisphere channels^43,44^. No significant difference was detected in intrahemispheric functional connectivity analysis within the AD group (p > 0.05, *uncorrected*).

#### HbR concentrations

Intrahemispheric adjacent channels of the channels showed significant effect across groups were determined. The new channel (ch.) pairs became interhemispheric adjacent channel pairs separately in the right and left hemispheres. Channels 15, 17 and 18 were located in the left hemisphere, and channels 37, 38 and 40 were located in the right hemisphere. Intrahemispheric adjacent channels of channel 15 are ch. 10, ch.11, ch.13, ch.14, ch. 16, ch. 17. Intrahemispheric adjacent channels of channel 37 are ch. 32, ch. 33, ch. 35, ch. 36, ch. 38, ch. 39. Intrahemispheric adjacent channels of channel 17 are ch. 10, ch. 11, ch. 15, ch. 18, ch. 19, ch. 20. Intrahemispheric adjacent channels of channel 38 are ch. 32, ch. 33, ch. 37, ch. 40, ch. 41, ch. 42. Intrahemispheric adjacent channels of channel 18 are ch. 16, ch. 17, ch. 19, ch. 20, ch. 21, ch. 22. Intrahemispheric adjacent channels of channel 40 are ch. 38, ch. 39, ch. 41, ch. 42, ch. 43, ch. 44.

The new channel pairs analyzed were as follows: 15–10 with 37–32 (1. adjacent ch. pairs), 15–11 with 37–33 (2. adjacent ch. pairs), 15–13 with 37–35 (3. adjacent ch. pairs), 15–14 with 37–36 (4. adjacent ch. pairs), 15–16 with 37–39 (5. adjacent ch. pairs), 15–17 with 37–38 (6. adjacent ch. pairs), 17–10 with 38–32 (7. adjacent ch. pairs), 17–11 with 38–33 (8. adjacent ch. pairs), 17–15 with 38–37 (9. adjacent ch. pairs), 17–18 with 38–40 (10. adjacent ch. pairs), 17–19 with 38–41 (11. adjacent ch. pairs), 17–20 with 38–42 (12. adjacent ch. pairs), 18–16 with 40–39 (13. adjacent ch. pairs), 18–17 with 40–38 (14. adjacent ch. pairs), 18–19 with 40–41 (15. adjacent ch. pairs), 18–20 with 40–42 (16. adjacent ch. pairs), 18–21 with 40–43 (17. adjacent ch. pairs), 18–22 with 40–44 (18. adjacent ch. pairs).

Paired sample t-test statistical testing was used to analyze functional connectivity differences between adjacent channel pairs within each hemisphere of both AD and control groups. The statistical results showed no significant difference in the functional connection analysis of parallel intrahemispheric adjacent channels in the two hemispheres in the control and AD groups (p > 0.05, *uncorrected*).

## Discussion

The current study investigates hemispheric asymmetry by comparing the hemodynamic responses obtained through functional connectivity analysis using fNIRS imaging between healthy older adults and those diagnosed with AD during resting state. Previous studies have shown that hemispheric asymmetry reduction increases with age^15,17,38^, which is described to as the HAROLD model^12^. The main objective was to compare the reduction of hemispheric asymmetry (if any) in healthy and Alzheimer's diagnosed older adults at resting-state fNIRS imaging and functional connectivity analysis.

First, we analyzed the functional connectivity between homologous brain regions and compared it between the two groups. Then, we determined the intrahemispheric adjacent channels of the channels showing significant differences and analyzed the intrahemispheric functional connectivity to compare the two hemispheres within the group.

The results indicate that the interhemispheric functional connectivity between homologous channels is stronger in the control group compared to AD group. This stronger functional connection suggests that two homologous brain regions are in greater communication, resulting in increased hemispheric symmetry. These findings suggest that the decrease in hemispheric asymmetry was more pronounced in the control group. In the AD group, interhemispheric functional connectivity is weaker than in the control group. This suggests that hemispheric asymmetry persists in the AD group. Some views have been proposed to explain the decrease in hemispheric asymmetry in the HAROLD model, including the compensation view. According to compensation view, the decrease in hemispheric asymmetry as we age is a compensatory mechanism for the negative effects of aging^12^. To evaluate this view, we compared subjects with AD, a neurodegenerative disease, to healthy elderly subjects. Our findings indicate that the decrease in hemispheric asymmetry was greater in the control group and less in the AD group. Additionally, we observed that the compensation mechanism was impaired in the neurodegenerative disease, whereas it was functioning in the healthy elderly. These results support the compensation view of the HAROLD model.

Roe et al.^10^ found that the cerebral cortex thins unevenly with age, particularly accelerated in AD. We link the age-related cortical thinning with declining functional hemispheric asymmetry. In healthy aging, reduced hemispheric asymmetry may compensate for asymmetric cortical thinning. However, in Alzheimer's, this compensation is less effective despite faster cortical thinning, suggesting an inability to offset the accelerated degeneration. Further research is needed to clarify the relationship between structural and functional changes and determine whether the diminished compensation in Alzheimer's is a cause or an effect of the disease.

According to Dolcos et al.'s right hemisphere aging hypothesis^3^, age-related cognitive declines affect right hemisphere functions more than left hemisphere functions. In line with this study, our study also revealed that, the functional connection was stronger in the left hemisphere in the control group. This indicates that the functional connection in the right hemisphere is weaker compared to the left hemisphere. Therefore, our study supports the right hemi-aging hypothesis. However, the analysis of intrahemispheric functional connectivity within the AD group did not reveal any significant differences. This means that both hemispheres in the AD group exhibited similar connectivity with the adjacent channels.

In the current study, significant differences were found in the channels based on interhemispheric functional connectivity analysis. Increased functional connectivity was found in the premotor (ch. 8 and 30) and supplementary motor areas (ch. 8 and 30), the primary motor areas (ch. 13 and 35/ch. 15 and 37), primary somatosensory areas (ch. 21 and 43/(ch. 15 and 37), the inferior parietal areas (ch. 21 and 43/ch.18 and 40), primary somatosensory areas (ch. 21 and 43) and supramarginal gyrus (ch. 17 and ch. 38). in healthy controls. This demonstrate us that the decrease in hemispheric asymmetry is greater in these regions in the control group. Our study is consistent with Agcaoolu et al.^39^, who found a decrease in resting-state lateralization with aging in the primary sensorimotor, premotor, supplementary motor and inferior parietal regions. Agcaoglu et al.^39^ also discovered that the lingual gyrus, attentional networks (inferior parietal lobule, superior parietal lobule, middle temporal gyrus), and frontal network (inferior frontal gyrus) become more symmetrical with aging. Although our results conflict with those of Zuo et al.^40^, who indicate a decrease in homotopic resting-state connectivity in the inferior parietal cortex, they are consistent with those of others^15,39^.

It is widely accepted that language functions are highly lateralized to the left hemisphere in right-handed individuals. Additionally, handedness has been found to be significantly associated with brain asymmetry^41^. Jin et al.^42^. evaluated passive vibrotactile perception and confirmed the relationship between handedness and hemispheric asymmetry. Our study assessed handedness using the Edinburgh Handedness Preference Inventory and found that all subjects were right-handed. Therefore, it is unsurprising that the analysis revealed stronger functional connections in the left hemisphere when comparing intrahemispheric adjacent channels with homologous channels in the control group. In right-handed subjects, the left hemisphere is predominantly used for motor performance. These asymmetries in the motor cortex have been interpreted as resulting from handedness^1,43,44^. Dinomais et al.^45^. found that the primary motor cortex showed leftward asymmetry, while the supplementary motor cortex showed rightward asymmetry in 30 healthy young right-handed subjects during a resting-state fMRI study. These asymmetries were detected by comparing the intrahemispheric functional connectivity analyzes of that region between the two hemispheres. In line with this previous study, our study also found a significant difference in functional connectivity analyses between interhemispheric homologous channels in the primary motor areas (channel 13 and 35).

Allen et al.^46^ found that the functional connection between the prefrontal cortex and hippocampus decreased in Alzheimer's patients, regardless of the hemisphere. These results shed light on our study, In our study, we found that interhemispheric functional connectivity was lower in the Alzheimer's group compared to the control group. This reduction in functional connectivity suggests a decrease in communication between most brain regions in Alzheimer's patients.

Cui et al.^47^ found that dynamic functional connectivity in the left precuneus decreased more in Alzheimer's patients. Love and Miners^48^ reported that there is functional and structural lateralization in the precuneus, and that Alzheimer's patients have more atrophy in the left precuneus compared to healthy aging. Furthermore, literature reports that functional loss and aging in Alzheimer's patients occur more rapidly in the left hemisphere, which is also responsible for asymmetry with aging^10,11^. Based on this evidence, our study's lower functional connectivity compared to the control group is a promising finding. However, we were unable to detect changes in functional connectivity in the right or left hemisphere of the Alzheimer group due to the lack of significant results in intrahemispheric functional connectivity.

Furthermore, we found that the primary motor cortex, which had previously shown left asymmetry in young healthy subjects^45^, exhibited increased functional connectivity between the two hemispheres and decreased intrahemispheric functional connectivity in the control group of healthy elderly subjects. Specifically, it was determined that there was a decrease in hemispheric asymmetry in the primary motor cortex region (ch. 13 and 35/ch. 15 and 37) in the control group compared to the AD group. Our findings contribute to the existing literature on the left hemispheric asymmetry of the primary motor cortex in young adults. We conclude that this hemispheric asymmetry is reduced when comparing older adults with the AD group.

Jaycay et al.^49^ examined functional connectivity in terms of modularity in young healthy control participants, most of whom were right-handed. They found increased modularity (or functional connectivity) in the left hemisphere, which is consistent with our study. Similarly, we detected increased functional connectivity in the control group during intrahemispheric functional connectivity analysis. Our control group consisted of older adults. The results suggest that the increase in functional connectivity in the left hemisphere remains stable with age. However, further investigation is required to examine intrahemispheric functional connectivity in Alzheimer's patients, with a larger sample size in the future.

Quaresima et al. and Scholkmann et al. have demonstrated that functional activation of the human cerebral cortex can also be successfully investigated using fNIRS, optical topography, NIR imaging, diffuse optical imaging (DOI), or diffuse optical tomography (DOT)^19,50^. Analyzing functional connectivity has some advantages in using resting-state data rather than data obtained during some tasks. During the performance of a task, external factors can clearly affect the connectivity between brain regions. In contrast, resting-state data can be analyzed from various perspectives, independent of specific inputs or external factors^43^. Zhang et al.^20^ investigated hemispheric asymmetry in young adults during the Stroop task and found that the left hemisphere exhibited greater functional connectivity than the right hemisphere, according to their analysis of NIRS data. Hemispheric asymmetry was identified through NIRS-based functional connectivity analysis. In a study by Medvedev et al.^28^, resting-state fNIRS data from 15 young subjects were analyzed, and it was found that resting-state functional connectivity demonstrated hemispheric asymmetry. It is worth noting that there are not many functional near-infrared spectroscopy (fNIRS) studies on resting-state activation in the literature^51^. We contributed to the literature by revealing the difference in hemispheric asymmetry between AD and control groups through functional connectivity analysis using the resting-state fNIRS imaging method.

In previous studies, resting-state brain image recordings typically lasted between 4 and 8 min^28,52–55^. Additionally, it should be noted that in some studies, recordings were conducted solely with open eyes^52,56^, while in others, recordings were conducted solely with closed eyes^54,55^. For our study, we analysed 4-min recordings with eyes open.

## Conclusion

Our study has demonstrated that functional connectivity can be measured using optical methods. One notable distinction of our study from existing literature is the comparison of hemispheric asymmetry between AD group and control group using resting-state functional connectivity analysis. Statistical comparisons revealed that functional connectivity increased in the premotor cortex (ch. 8 and ch. 30), supplementary motor cortex (ch. 8 and ch. 30), primary motor cortex (ch. 13 and ch. 35/ch. 15 and ch. 37), inferior parietal cortex (ch. 21 and ch. 43/(ch. 18 and ch. 40), primary somatosensory cortex (ch. 21 and ch. 43)/(ch. 15 and ch. 37), and supramarginal gyrus (ch. 17 and ch. 38), regions in the control group compared to the AD group. It has been found that hemispheric asymmetry decreases more in the control group than in Alzheimer's patients. In line with our results, we have demonstrated that alterations in functional connectivity can be identified during the resting-state with the fNIRS method, and we think that these are results that will guide neuromodulation treatments in the clinic.

## Limitations

The study was limited by the number of participants, as only 14 AD and 16 control group subjects were analyzed using fNIRS data. More meaningful statistical results could have been obtained with larger subject groups. Another factor that limited our study was the analysis of 4-min data. It was a difficult experiment for Alzheimer's patients to wait without moving and focusing for a long time. Therefore, the data obtained for a maximum of 4 min was analyzed. Additionally, there was no group of healthy young adults in our study. The results will be more convincing if we include a test group including healthy young adults in the future. In our study, age and sex were not equal across groups, so we performed regression analysis to assess the relationship between age, sex and functional connectivity. Regression analysis showed that age and sex did not have a critical significant effect on our results, but further studies with patient and control groups of equal age and sex are needed.

### Supplementary Information

Supplementary Table S1.

## Data Availability

The datasets utilized and/or analyzed in the present study are available from the corresponding author upon reasonable request.
