# Supplementary material for: Investigation of hemispheric asymmetry in Alzheimer’s disease patients during resting state revealed by fNIRS
Source: Sci Rep. 2024 Jun 11;14:13454. doi: 10.1038/s41598-024-62281-y (PMC11166983; doi:10.1038/s41598-024-62281-y)
Supplement: Supplementary file 1 — Supplementary Table S1. [file 41598_2024_62281_MOESM1_ESM.docx]

**INVESTIGATION OF HEMISPHERIC ASYMMETRY IN ALZHEIMER’S DISEASE PATIENTS DURING RESTING STATE REVEALED BY fNIRS**

Hazel Gül Mızrak ^1^ (hazel.mizrak@medipol.edu.tr)

Merve Dikmen ^2,3^ (merve.dikmen@medipol.edu.tr)

Lütfü Hanoğlu ^2,4^ (lhanoglu@medipol.edu.tr)

Bayram Ufuk Şakul ^1^ ([usakul@medipol.edu.tr](mailto:usakul@medipol.edu.tr))

1. Department of Anatomy, School of Medicine, Istanbul Medipol University, Istanbul, Turkey

2. Regenerative and Restorative Medicine Research Center (REMER), Research Institute for Health Sciences and Technologies (SABITA), Istanbul Medipol University, Istanbul, Turkey

3. Program of Electroneurophysiology, Vocational School of Health Services, Istanbul Medipol University, Istanbul, Turkey

4. Department of Neurology, Istanbul Medipol University Training and Research Hospital, Istanbul, Turkey

**Correspondence:**

Dr. Merve Dikmen

PhD of Neuroscience Department

Istanbul Medipol University Regenerative and Restorative Medicine Research Center (REMER), Research Institute for Health Sciences and Technologies (SABITA)

Göztepe mah. Atatürk cad. No:40, Beykoz/ISTANBUL

Email: merve.dikmen@medipol.edu.tr

**CHANNELS AND ANATOMICAL LOCATION**

| **Left and right Channel (Ch.)** | **Anatomical location** |
| --- | --- |
| 1st and 23rd Ch. | Dorsolateral prefrontal cortex |
| 2nd and 24th Ch. | Temporopolar area (BA) |
| 3rd and 25th Ch. | Dorsolateral prefrontal cortex |
| 4th and 26th Ch. | Superior medial frontal area |
| 5th and 27th Ch. | Middle frontal gyrus |
| 6th and 29th Ch. | Middle frontal gyrus |
| 7th and 28th Ch. | Triangular part of inferior frontal gyrus |
| 8th and 30th Ch. ** | Premotor and supplementar motor area |
| 9th and 31st Ch. | Opercular part of inferior frontal gyrus |
| 10th and 32nd Ch. | Subcentral area (BA) |
| 11th and 33rd Ch. | Middle temporal gyrus |
| 12th and 34th Ch. | Dorsolateral prefrontal cortex |
| 13th and 35th Ch. ** | Primary motor cortex |
| 14th and 36th Ch. | Primary motor cortex |
| 15th and 37th Ch. * | Supramarginal gyrus |
| 16th and 39th Ch. | Primary somatosensorial cortex |
| 17th and 38th Ch. * | Superior temporal gyrus |
| 18th and 40th Ch. * | Inferior parietal gyrus  Supramarginal gyrus |
| 19th and 41st Ch. | Middle temporal gyrus |
| 20th and 42nd Ch. | Middle temporal gyrus |
| 21st and 43rd Ch. ** | Inferior parietal cortex  Primary somatosensorial cortex |
| 22nd and 44th Ch. | Angular gyrus  Inferior parietal korteks |
| 45th and 47th Ch. | Frontal eye fields (BA) |
| 46th and 48th Ch. | Frontal eye fields (BA)  Dorsolateral prefrontal cortex |

**Supplementary Table S1:** Table of functional near-infrared spectroscopy (fNIRS) channels and their corresponding anatomical locations.

The anatomical locations were determined using the AAL2 brain atlas through the fOLD toolbox(16). The Broadmann area was identified as BA.

**: Significant brain areas of interhemispheric connectivity between groups in HbO concentrations.

*: Significant brain areas of interhemispheric connectivity between groups in HbR concentrations.
